# Role of UDP-Glucuronosyltransferase 1A1 in the Metabolism and Pharmacokinetics of Silymarin Flavonolignans in Patients with HCV and NAFLD

**DOI:** 10.3390/molecules22010142

**Published:** 2017-01-15

**Authors:** Ying Xie, Sonia R. Miranda, Janelle M. Hoskins, Roy L. Hawke

**Affiliations:** 1 Division of Pharmacotherapy and Experimental Therapeutics, UNC Eshelman School of Pharmacy, University of North Carolina at Chapel Hill, Chapel Hill, NC 27599, USA; yxie@must.edu.mo (Y.X.); mirandasr@gmail.com (S.R.M.); janelle.hoskins1@gmail.com (J.M.H.); 2 State Key Laboratory for Quality Research of Chinese Medicines, Macau University of Science and Technology, Avenida Wai Long, Taipa, Macau, China

**Keywords:** UGT1A1, polymorphism, pharmacokinetics, silymarin, silybin A, liver disease, NAFLD, HCV

## Abstract

Silymarin is the most commonly used herbal medicine by patients with chronic liver disease. Silymarin flavonolignans undergo rapid first-pass metabolism primarily by glucuronidation. The aims of this investigation were: (1) to determine the association of UGT1A1*28 polymorphism with the area under the plasma concentration-time curves (AUCs) for silybin A (SA) and silybin B (SB); (2) to evaluate the effect of UGT1A1*28 polymorphism on the profile of flavonolignan glucuronide conjugates found in the plasma; and (3) to investigate the role of UGT1A1 enzyme kinetics on the pharmacokinetics of SA and SB. AUCs and metabolic ratios for thirty-three patients with chronic liver disease administered oral doses of silymarin were compared between different UGT1A1*28 genotypes. The AUCs, metabolic ratios, and the profiles of major SA and SB glucuronides did not differ significantly among the three *UGT1A1* genotypes. In contrast, an increase in the proportion of sulfated flavonolignan conjugates in plasma was observed in subjects with UGT1A1*28/*28 genotype compared to subjects carrying wild type alleles. Differences in SA and SB in vitro intrinsic clearance estimates for UGTIA1 correlated inversely with SA and SB exposures observed in vivo indicating a major role for UGT1A1 in silymarin metabolism. In addition, a significant difference in the metabolic ratio observed between patients with NAFLD and HCV suggests that any effect of *UGT1A1* polymorphism may be obscured by a greater effect of liver disease on the pharmacokinetics of silymarin. Taken together, these results suggest the presence of the UGT1A1*28 allele does not contribute significantly to a large inter-subject variability in the pharmacokinetics of silybin A and silybin B which may obscure the ability to detect beneficial effects of silymarin in patients with liver disease.

## 1. Introduction

Silymarin, a purified extract from milk thistle (*Silybum marianum*) and mainly composed of six flavonolignans including silychristin, silydianin, silybin A, silybin B, isosilybin A and isosilybin B [1,2], is the most commonly used herbal product by individuals with chronic liver disease. In chronic hepatitis C virus (HCV) infection, approximately one-third of patients were found to have either previously, or were currently, self-treating their liver disease with silymarin [3]. The antioxidant, anti-inflammatory, and antifibrotic properties of silymarin have been demonstrated in various animal and in vitro models [1,4]. In addition, silymarin has been shown to possess significant anti-viral effects against HCV infection with mechanism of suppression of HCV NS5B RNA dependent RNA polymerase activity, inhibition of inflammatory cytokines and hepatocyte NF-κB signaling [5,6,7].

Silymarin may be one of the most potent antioxidants found in nature by virtue of its free radical scavenger reactivity and favorable membrane-lipid/water partitioning [8]. Oxidative stress is thought to play a central role in the etiology of nonalcoholic steatohepatitis (NASH), a specific subset of nonalcoholic fatty liver disease (NAFLD), and is hypothesized to represent a ‘second hit’ triggering the necroinflammatory response characteristic of NASH [9]. Therefore, the antioxidant properties of silymarin may be particularly beneficial as a treatment for NASH since patients have significantly increased levels of serum lipid peroxidation products as well as other oxidative stress markers and decreased levels of antioxidant enzymes [10,11]. In addition, oxidative stress is a key feature of disease activity in HCV infection. Elevated levels of oxidative stress markers have been associated with the grade and stage of liver disease in patients with HCV which suggests that antioxidant therapy may be effective in slowing disease progression in the absence of antiviral effects [12]. These observations provide the rationale for current Phase 2 trials on the effects of silymarin in HCV and NASH populations. Although initially evaluated for its hepatoprotective properties, silymarin has recently been shown to demonstrate additional beneficial properties in a number of different human disease states including cardiovascular, diabetes, cancer, and Alzheimer’s [13].

However, in a large, robust randomized clinical trial, higher than customary doses of silymarin did not significantly alter biochemical or virological markers of disease activity in patients with chronic HCV [14]. Low oral bioavailability and large inter-individual variability in silymarin plasma exposures observed in patients with chronic HCV may account for the inability to establish the efficacy of silymarin [15]. Various pharmaceutical formulations of silymarin have been developed to improve oral bioavailability through increased absorption and chemical stability through phospholipid complexation, and nanoparticle and liposomal delivery [16]. Alternatively, since a large number of clinical studies, including those described above, have been conducted using a standardized silymarin product with uniform bioavailability (Legalon^®^), we hypothesize that the large inter-individual variation noted in these studies may reflect patient-specific factors influencing silymarin disposition through effects on absorption, distribution, metabolism, or elimination.

Silybin A (SA) and silybin B (SB) are the predominant silymarin flavonolignans found in human plasma following oral administration of silymarin. Silymarin flavonolignans undergo rapid and extensive phase II conjugative metabolism followed by primarily biliary excretion, and are mainly present as glucuronides and sulfates in human plasma [15]. Therefore, metabolism by UDP-glucuronosyltransferases (UGT) and sulfotransferases (SULT) appear to be major pathways in the metabolism of silymarin. While in vitro investigations have suggested a major role for UGT1A1 in the glucuronidation of SA and SB [17,18], the importance of UGT1A1-mediated glucuronidation on the metabolism and pharmacokinetics of silymarin flavonolignans has not been investigated in humans.

UGT1A1 is the major *UGT1* gene product and numerous polymorphisms have been identified [19]. The UGT1A1*28 polymorphism is a common genetic variant with reduced glucuronidation activity and is characterized by an extra thymine-adenine (TA) repeat in the promoter region of the gene compared with the wild-type allele, UGT1A1*1, which has 6 TA repeats [13,20]. Bilirubin is an endogenous substrate for UGT1A1 and Gilbert syndrome is characterized by abnormally high levels of circulating unconjugated bilirubin due to reduced UGT1A1 activity in carriers of the UGT1A1*28 allele [14,21]. Altered pharmacokinetics due to the functional deficiency of UGT1A1 is also expected for drugs metabolized predominately by UGT1A1. Clinical studies have demonstrated a significant correlation between the UGT1A1*28 polymorphism and the pharmacokinetics of drugs [22,23,24]. Thus, differences in *UGT1A1* genotypes may contribute to the variability in silymarin’s pharmacokinetics and to the inconsistencies in clinical outcomes that have been observed [18,19,20,25,26,27].

The purpose of this study was to investigate the role of *UGT1A1* polymorphism on the metabolism and pharmacokinetics of silymarin flavonolignans in patients with chronic HCV infection or nonalcoholic fatty liver disease (NAFLD). We evaluated the association of the pharmacokinetics of SA and SB and the UGT1A1*28 polymorphism as well as the UGT1A1 enzyme kinetics in vitro. Additionally, we evaluated the effect of the UGT1A1*28 polymorphism on the pattern of major glucuronide conjugates of silymarin flavonolignans found in the patient plasma, as well as on the proportion of glucuronides or sulfates in total conjugated metabolites.

## 2. Results

### 2.1. Enzyme Kinetics of Silymarin Glucuronidation in Recombinant Human UGT1A1 by Substrate Depletion Assay

SA, SB, and silydianin were selected for in vitro UGT1A1 enzyme kinetic studies since previous clinical investigations showed these were the major silymarin flavonolignans found in plasma from patients with HCV infection following oral dosing [15]. UGT1A1 Michaelis–Menten kinetic parameters using either SA, SB, or silydianin as substrates are depicted in Table 1. As expected, the apparent *K_m_* values obtained using SA, SB, and silydianin as substrates were within approximately 2-fold of the IC_50_ values we have previously reported for flavonolignan inhibition of UGT1A1 catalyzed glucuronidation of the probe substrate, 7-hydroxy-4-trifluoromethylcoumarin (7-HFC), when incubated at 7-HFC’s *K_m_* concentration [18]. The observed higher rates for SB and silydianin glucuronidation (*V_max_* > 5900), their lower *K_m_* (~9 µM), and overall higher intrinsic clearances (691 and 1411, respectively) suggest UGT1A1 is likely a predominant UGT isoform for the metabolism of SB and silydianin compared to SA.

### 2.2. Influence UGT1A1*28 Polymorphism on SA and SB Plasma Exposures

Baseline demographics and disease characteristics for patients with HCV or NAFLD were comparable across all genotyping groups (Table 2). Homozygous *28/*28, heterozygous *1/*28, and wild-type *1/*1 genotype frequencies were 15.2%, 42.4%, and 30.3%, respectively. Allelic frequencies for *1(TA6) and *28(TA7) were 52% and 42%, respectively.

**Figure 1:**
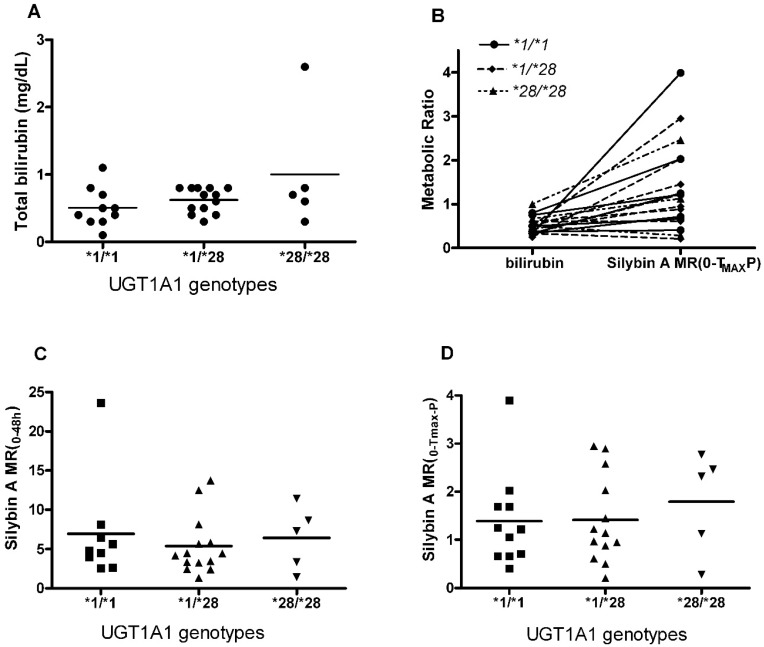
Bilirubin concentrations and silybin A metabolic ratios in different *UGT1A1* promoter genotype groups. (**A**) Total bilirubin concentration in hepatitis C virus (HCV) and nonalcoholic fatty liver disease (NAFLD) patients with different *UGT1A1* genotypes; (**B**) Relationship between silybin A (SA) metabolic ratio (MR) _(0-Tmax-P)_ and ratio of conjugated bilirubin to total bilirubin; (**C**,**D**) SA MR _(0–48 h)_ and MR _(0-Tmax-P)_ with different *UGT1A1* genotypes.

SA and SB were the only flavonolignans quantified in patient plasma in all dose cohorts. As depicted in Table 3, no significant differences were observed in the AUC_0–48 h_ for either SA or SB among *UGT1A1* genotypes. Because parent flavonolignans are extensively metabolized to varying degrees, which might mask an effect of *UTG1A1* genotype on silymarin disposition, the AUC ratio for total conjugates to parent flavonolignan, MR_0–48 h_, was also evaluated. In addition, because flavonolignan conjugates under extensive enterohepatic cycling which might also mask the effect of UGT1A1 genotype, we also evaluated the AUC ratio of total conjugates to parent flavonolignan from time 0 to peak concentration, MR_0-Tmax-P_, for each flavonolignan. Mean MR_0–48 h_ and MR_0-Tmax-P_ ratios for SA (Figure 1C,D, respectively) and SB (data not shown) did not differ among UGT1A1 *1/*1, *1/*28, and *28/*28 genotypes.

Individuals with the homozygous *28/*28 genotype have significantly higher serum bilirubin concentrations than those with genotypes *1/*28 and *1/*1 [28]. However, the relationship between *UGT1A1* polymorphism and bilirubin concentration in patients with liver disease has not been previously examined. In this study, a similar trend towards higher serum total and direct bilirubin concentration was observed for *28/*28 genotype (Figure 1A), however no statistically significant association was observed between serum total bilirubin concentration and *UGT1A1* genotypes (Figure 1B) in patients with HCV or NAFLD suggesting that the effect of HCV liver disease may mask the influence of genotype that has been observed in other patient populations.

### 2.3. Effect of Liver Disease Type on Metabolic Ratios for SA and SB

**Figure 2:**
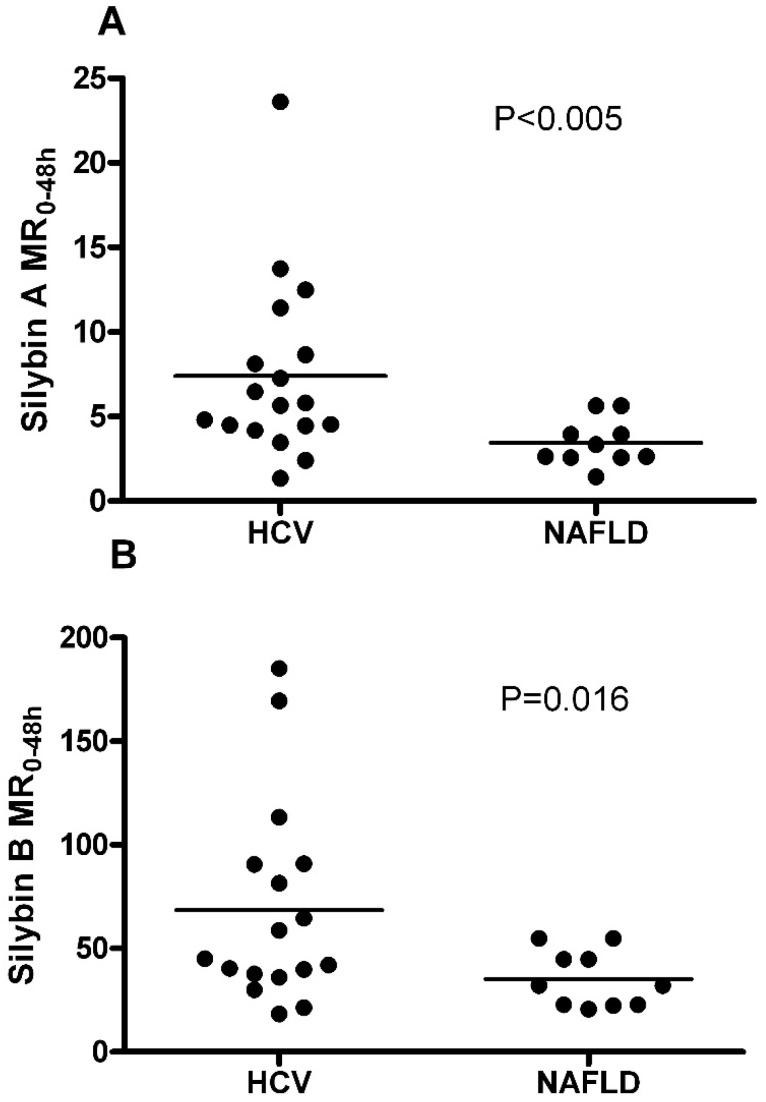
Silybin (**A**,**B**) metabolic ratio (MR) stratified by liver disease type. Mean MR in patients with HCV infection and NAFLD were 7.4 and 4.5 for SA (*p* < 0.005), and 68.6 and 43.4 for silybin B (SB) (*p* = 0.016), respectively, using student’s *t*-test.

Differences in the disposition of silymarin between patients with different types and stages of liver disease compared with healthy volunteers has been reported [29]. To determine whether our analysis of the association of metabolic ratios with the UGT1A1*28 polymorphism might be confounded by differences due to liver disease types, the mean metabolic ratio, MR_(0–48 h)_, between HCV and NAFLD subjects were compared. Mean values for HCV and NAFLD subjects were 7.4 and 4.5 for SA (*p* < 0.005), and 68.6 and 43.4 for SB (*p* < 0.05), respectively (Figure 2). These data suggest liver disease has a significant effect on the pharmacokinetics of silymarin.

### 2.4. Metabolites Profile of Silymarin and UGT1A1*28 Effects

Silymarin flavonolignans are metabolized primarily to sulfate and glucuronide conjugates [16,30]. We hypothesized reduced glucuronidation activity associated with UGT1A1*28 alleles may result in a change in the amounts of individual glucuronides but not in their total amount due to the contribution of other UGTs, or in a shift competing metabolic pathways, such as sulfation, could potentially compensate for the reduced glucuronidation function in patients with UGT1A1*28 alleles. Therefore, patients with UGT1A1*28 alleles may have higher percentage of sulfates and lower percentages of glucuronides of silymarin in metabolic pattern but have similar total metabolites ratio as wild type.

**Figure 3:**
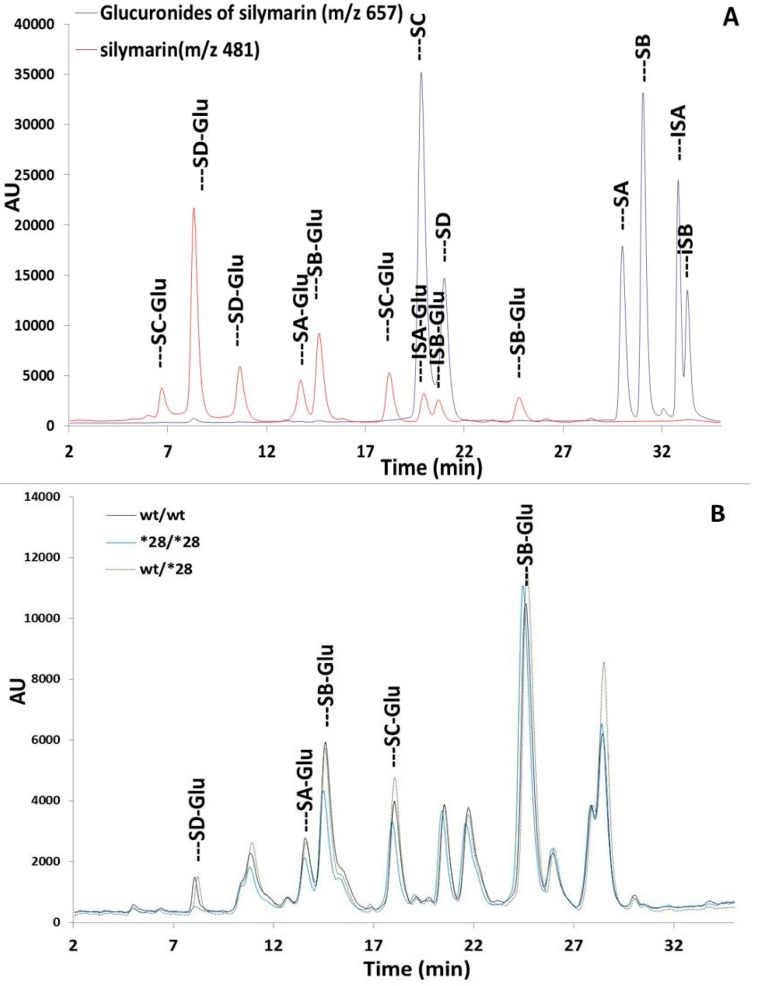
Chromatographic profiles of flavonolignan glucuronide conjugates. (**A**) Composite profile of silymarin flavonolignans and their identified glucuronide conjugates following the incubation of individual flavonolignans with UGT1A1; (**B**) Comparison of chromatographic profiles of identified flavonolignan glucuronide conjugates form pooled plasma from patients with different UGT1A1 genotypes.

To better understand the UGT1A1*28 effects on the metabolites profile of six silymarin flavonolignans, we developed a liquid chromatography-mass spectrometry (LC-MS) method to investigate the glucuronide conjugates of silymarin flavonolignans in plasma. Silymarin flavonolignans are rather complex targets for conjugation with five hydroxyl groups, resulting in the formation of a multitude of conjugates in vivo. Further complexity arises from the occurrence of silymarin in six isomers forms with same molecular weight. A gradient elution program was optimized for good separation of all glucuronides for each of the six isomers after incubation with recombinant UGT1A1 or HLM using LC-ESI-MS (Figure 3A).

**Figure 4:**
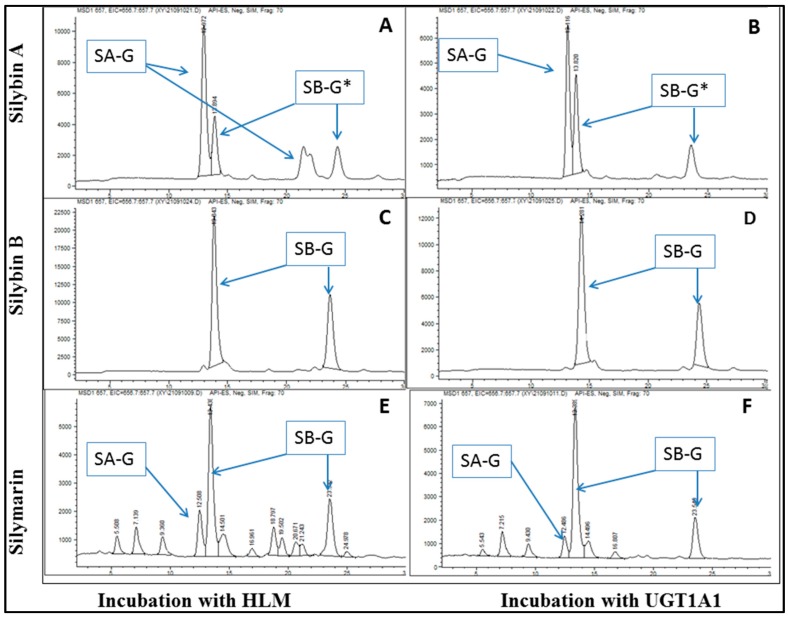
Typical chromatograms of silymarin flavonolignans and their glucuronides generated in vitro by incubation with human liver microsome (HLM) (**A**,**C**,**E**) or UGT1A1 (**B**,**D**,**F**). (**A**,**B**): SA was incubated with HLM (**A**) or UGT1A1 (**B**). * SB glucuronide peaks are present because there are small amounts of SB in SA standard solution; (**C**,**D**): SB was incubated with HLM (**C**) or UGT1A1 (**D**); (**E**,**F**): Silymarin mixture was incubated with HLM (**E**) or UGT1A1 (**F**). Incubation with HLM or UGT1A1 and liquid chromatography-mass spectrometry analysis were performed as described under *Materials and Methods*.

Glucuronides of each silymarin flavonolignan were biosynthesized by their incubation with human liver microsomes (HLM) or recombinant UGT1A1 and were confirmed by mass spectrometry (Figure 3A). These studies showed that the major glucuronides formed using either HLM or recombinant UGT1A1 were mono-glucuronides. Isosilybin B was metabolized to only on glucuronide species; while both SB and silydianin formed two major glucuronides; silychristin was metabolized to four glucuronides and isosilybin A to three glucuronides species (Figure 3A). A similar metabolite profile was observed after silymarin was incubated with HLM and UGT1A1 (Figure 4), which suggests UGT1A1 may be the predominant UGT isozymes for the glucuronidation of silymarin. In contrast, SA formed one glucuronide following incubation with UGT1A1 (Figure 4B), however, more than one glucuronide was observed following SA incubation with HLM (Figure 4A). In addition, peaks corresponding to silybin B glucuronides were observed in the incubation mixture following the incubation of SA with either HLM or UGT1A1 which was due to a small amount of SB in the SA standard solution (Figure 4A,B).

Identification of glucuronides in plasma from subjects was based on their chromatographic retentions and MS in-source fragmentations, and was further confirmed by adding biosynthesized glucuronides of each individual isomer to plasma samples (Figure 3B). Five glucuronides were identified with retention time of 7.3 min (SD-Glu), 13.0 min (SA-Glu), 17.6 min (SC-Glu), 13.9 min (SB-7-β-Glu), and 24.0 min (SB-20-β-Glu) in Figure 3B. Patient plasma samples near T_max_ are pooled separately based on their *UGT1A1* genotypes. With equal amount of total silymarin in each pooled sample, the metabolites profiles were same for all *UGT1A1* genotypes (Figure 3B). Thus, the UGT1A1*28 allele had no effect on the metabolite profiles of silymarin flavonolignans.

**Figure 5:**
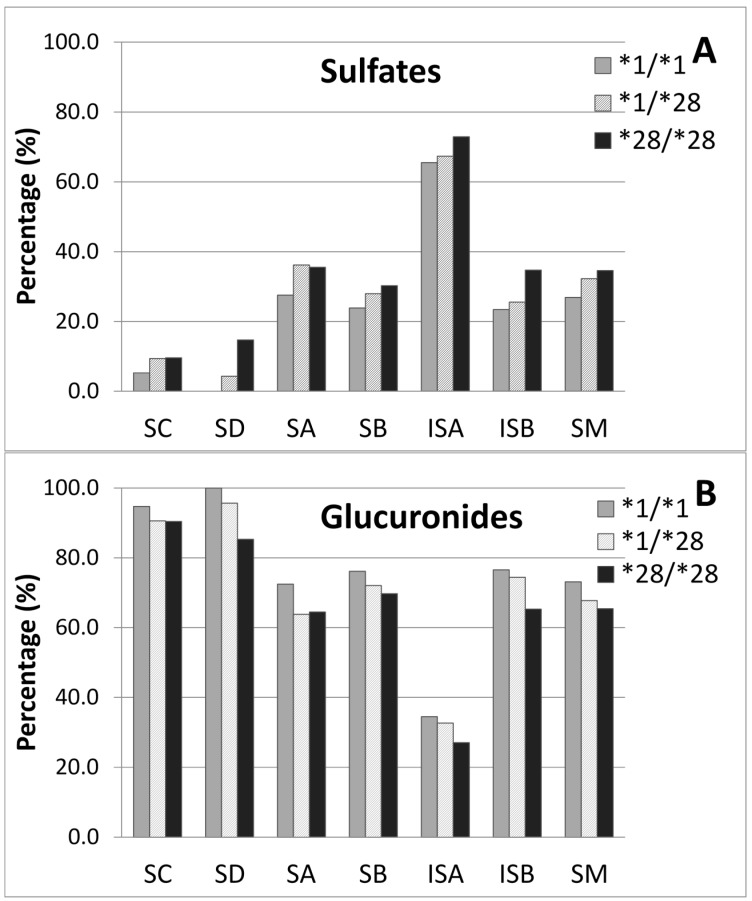
The influence of UGT1A1*28 allele on the percentage of (**A**) sulfate or (**B**) glucuronide conjugates of silymarin flavonolignans in pooled patients’ plasma at 2 h post-dose. Open bar represents UGT1A1 *1/*1, hatched bar for *1/*28 and closed bar for *28/*28.

The sulfates and glucuronides of silymarin flavonolignans were indirectly determined with enzyme hydrolysis using sulfatase and β-glucuronidase, respectively. As seen in Figure 5, there was a slight increase in the percentage of sulfates and decrease in the percentage of glucuronides in the pooled plasma sample with UGT1A1*28 alleles compared to wild types. The mean percentages of sulfated and glucuronidated silymarin flavonolignans were 34.6% and 65.4% for homozygous, 32.2% and 67.8% for heterozygous, and 26.9% and 73.1% for wild type, respectively. After oral administration, all sulfate and glucuronide conjugates in patient plasma for *1/*28 and *1/*1 genotypes accounted for 83% of the total silymarin measured at the plasma at T_max_ and 80% in patient plasm of *28/*28 genotypes which is similar to what has been reported in healthy volunteers [30]).

Thus, there were no significant differences in the metabolites profiles of patients’ plasma with different *UGT1A1* genotypes (Figure 3B) as well as the conjugation rate of sulfates or glucuronides of each flavonolignan (Figure 5). Consequently, *UGT1A1* polymorphisms do not appear to affect the metabolism of silymarin flavonolignans in patients with HCV or NAFLD.

## 3. Materials and Methods

### 3.1. Subjects and Study Design

This study is a secondary analysis of prospectively collected data from NIH-sponsored Phase 1 clinical trial conducted by the SyNCH consortium (ClinicalTrials.gov Identifier: NCT00389376). This trial enrolled 56 subjects diagnosed with either HCV or NAFLD who were randomized into seven groups and treated with dosages of 140, 280, 560, and 700 mg of silymarin. Details of inclusion and exclusion criteria, subjects’ information and study design have been published [15]. All subjects provided written informed consent with optional participation for DNA collection, and the studies were approved by the Institutional Review Board. Of the fifty-six subjects from the Phase 1 trial, twenty-three were excluded from this analysis for lack of genetic information. Therefore, thirty-three subjects were eligible for inclusion in this study.

### 3.2. Chemicals and Reagents

Standardized silymarin, silybin (48% SA and 52% SB), β-glucuronidase (type B-10 from bovine liver), β-glucuronidase (from *Escherichia coli*), sulfatase (type H-1 from Helix pomatia), and naringenin (NG) were purchased from Sigma-Aldrich (St. Louis, MO, USA). Ultra Pool human liver microsomes (150 donors) and recombinant UGT1A1 were purchased from BD Biosciences (Bedford, MA, USA). Reference standards of silychristin and silydianin were purchased from ChromaDex (Santa Ana, CA, USA) and U.S. Pharmacopoeia (Rockville, MD, USA), respectively. Reference standards of SA and SB, isosilybin A and isosilybin B were obtained as a generous gift from Ulrich Mengs (Madaus GmbH, Cologne, Germany). The purity of all six silymarin flavonolignan standards was between 97% and 99%. All other chemicals and reagents were of analytical grade or higher and were purchased from commercial sources.

Silymarin (Legalon^®^, Lot No. 0418901) and matching placebo were manufactured in hard capsules by Madaus Rottapharm Group (Cologne, Germany). The flavonolignan content of each capsule was 23.2 mg, silybin A; 32.0 mg, silybin B; 11.8 mg, isosilybin A; 6.6 mg, isosilybin B; 24.9 mg, silychristin; and 29.0 mg, silydianin [21]. These six flavonolignans account for 70.8% (127.5 mg silymarin equivalent to 140 mg of silymarin) of the 180 mg milk thistle extract contained in each capsule.

### 3.3. LC/MS Conditions

The analysis of silymarin flavonolignans and their major glucuronidates, and total sulfates was performed on an Agilent HP 1100 LC-MS system (Agilent, Santa Clara, CA, USA). A C_18_ analytical column (Atlantis T3, 50 × 2.1 mm i.d., 3 μm particle size, Waters Corp., Ireland) was used for detection with a C_18_ Security Guard cartridge (4 × 2.0 mm i.d.; Phenomenex, Torrance, CA, USA). Glucuronides of silymarin were well separated using optimized gradient elution with methanol/0.5% acetic acid (pH 4) as mobile phase at a flow rate of 0.3 mL/min with a run time of 45 min. The gradient employed to obtain the conjugation profiles was as follows (mobile phase B: methanol): 0 min: 25%, 23 min: 40%, 30 min: 55%, 40 min: 70%, 41 min: 25%. Acetic acid was selected as an additive rather than trifluoroacetic acid or formic acid because it provided the most abundant deprotonated molecular ions [M − H]^−^ characteristic for silymarin flavonolignans under negative ESI.

MS parameters: capillary voltage, −3500 V; nebulizer pressure, 30 psi; drying gas, 8 L/min; drying gas temperature, 350 °C; fragment voltage, 70 V; dwell time, 100 ms; scan mode, selective ion monitoring (SIM) with [M − H]^−^ for silymarin flavonolignans (*m*/*z* 481), mono-glucuronide (*m*/*z* 657), mono-sulfate (*m*/*z* 561), and NG (*m*/*z* 271), respectively. LC-MS data were obtained by Agilent ChemStation Software.

The limit of quantification and linear quantitative range for the six silymarin flavonolignans was 5 ng/mL, and 5 to 1000 ng/mL, respectively. Accuracy, intraday and inter-day precisions (*n* = 5) for each silymarin flavonolignan was 95.4% to 107.4%, 1.7% to 11% and 4.5% to 14%, respectively.

### 3.4. UGT Genotyping

*UGTIA1* genotyping was performed on blood samples obtained from the 33 eligible subjects using pyrosequencing methods and primers which have been previously described [23]. Only carriers of the wild-type and *28 alleles were included in the analysis. Two subjects were carriers of the UGT1A1*36 alleles and two subjects were carriers of the UGT1A1*37 alleles. Therefore, only twenty-nine subjects (19 HCV and 10 NAFLD) were available to examine the association between *UGT1A1* polymorphism and the pharmacokinetics of SA and SB.

### 3.5. UGT1A1 Substrate Depletion Assay

In substrate depletion experiments, silydianin, SA, and SB with different concentrations from 0 to 40 μM (in 0.5 µL of DMSO with final percentage of 0.05% in incubation solution) were incubated with recombinant UGT1A1 (0.25 mg/mL). The reaction was initiated by the addition of 5 mM uridine 5’-diphospho-glucuronic acid (UDPGA); blank incubations without UDPGA were used as negative controls. Aliquots (100 µL) of the incubation mixture were removed at 90 min (based on the time linearity experiment) and the reaction terminated by the addition of 100 µL of ice-cold acetonitrile/glacial acetic acid (96/4, *v*/*v*) containing internal standard (NG). After the removal of protein by centrifugation at 15,000× *g* for 15 min at 4 °C, the supernatants were evaporated, and treated as described in Section 3.6 below, and then 25 µL of the final reconstituted samples was analyzed by LC-ESI-MS. Apparent *K_m_* and *V_max_* values for each substrate were estimated using the E_max_ model within Phoenix^®^ WinNonlin^®^ 5.2 (Certara, L.P., 1699 S Hanley Road, St Louis, MO, USA). The intrinsic clearance was calculated by *K_m_*/*V_max_*.

### 3.6. Biosynthesis of Glucuronides for Each Silymarin Flavonolignan

Glucuronides of each silymarin flavonolignan were biosynthesized using pooled human liver microsomes (HLMs) or recombinant UGT1A1 under the following conditions: 0.5 mg/mL protein, 25 µg of alamethicin/mg protein, 50 µM of each silymarin flavonolignan (in 1% dimethyl sulfoxide), 5 mM UDPGA, and 5 mM MgCl_2_ in 100 mM Tris buffer (pH 7.4). Reactions were carried out at 37 °C in a shaking water bath and terminated with the addition of 100 μL of ice-cold acetonitrile containing 1% glacial acetic acid and internal standard (NG, 40 ng) after 30 min. Mixtures were vortexed and centrifuged at 15,000× *g* for 15 min at 4 °C to precipitate protein. Supernatants were transferred to fresh tubes and dried completely under nitrogen. The residue was reconstituted with 100 µL mobile phase and well vortexed for ~2 min. Each sample was then transferred to a 1.5 mL centrifuge tube and centrifuged at 15,000× *g* at 4 °C for 15 min. A 25 µL aliquot of the centrifuged supernatant was injected into the LC-MS system. Generated glucuronides for each silymarin were used to develop the LC-MS method.

### 3.7. Quantification of Total Glucuronide and Sulfate Concentrations in Pooled Plasma Samples

All plasma samples were previously collected, quantified, and stored at −80 °C as previously described [8]. In the present study, patients’ plasma samples at the time of T_max_ were thawed on ice and pooled into three groups according to genotype (wild-type UGT1A1*1/*1, heterozygous *1/*28 and homozygous *28/*28). Based on the total flavonolignan concentration in individual samples, equal amounts of total (i.e., parent + conjugates) flavonolignans were mixed together to obtain pooled plasma samples for each genotype. These pooled plasma samples were used to compare the glucuronide pattern for silymarin flavonolignans in patients with different *UGT1A1* genotypes.

To estimate the concentration of total glucuronide and sulfate conjugates of silymarin flavonolignans in plasma, enzymatic hydrolysis with glucuronidase only from *Escherichia coli* (32,000 U/mL), sulfatase only with d-saccharic acid 1, 4-lactone (20 mM), a specific β-glucuronidase inhibitor to avoid the potential for simultaneous cleavage of glucuronides during desulfation, or both using bovine liver (8000 U/mL) and sulfatase (80 U/mL) was performed with 100 µL aliquots of pooled samples. Completion of the deconjugation reaction was monitored by mass spectrometry. In parallel, 100 µL aliquots of plasma without enzymes were treated with 400 µL of ice-cold acetonitrile containing 1% glacial acetic acid and NG (40 ng) to determine the concentration of parent silymarin flavonolignans in plasma. Hydrolysis reactions with β-glucuronidase from *Escherichia coli* were buffered by 0.4 M phosphate buffer (pH 7.5), while other incubations were buffered with 0.25 M sodium acetate (pH 5.0). Plasma samples with the different hydrolytic enzymes were incubated at 37 °C with gentle shaking overnight or at least 8 h. The reactions were terminated by the addition of 400 µL of ice-cold acetonitrile containing 1% glacial acetic acid and internal standard (NG, 40 ng). After the removal of protein by centrifugation at 15,000× *g* for 15 min at 4 °C, the supernatants were evaporated, and treated as described in Section 3.6 above. The total concentrations of glucuronides and sulfates in samples were estimated from the difference between the amount of silymarin flavonolignans released by incubation with both glucuronidase and sulfatase and the amount of silymarin flavonolignans released by either sulfatase or glucuronidase alone.

To obtain a metabolites profiles of the major glucuronides in plasma, 100 µL aliquots of pooled samples were treated with the mixture of sulfatase (160 U/mL) and a specific β-glucuronidase inhibitor, d-saccharic acid 1,4-lactone (20 mM). Direct monitoring of the pattern of major glucuronides was performed using selective ion monitoring for mono-glucuronides (*m/z* 657), and mono-sulfates (*m/z* 561) as described above.

### 3.8. Pharmacokinetics Analysis

Areas under the plasma concentration-time curves from time 0 to 48 h (AUC_0–48 h_); maximum plasma concentration (C_max_); and time to C_max_ (T_max_) were calculated using noncompartmental methods with Phoenix^®^ WinNonlin^®^ 5.2 (Certara, L.P., 1699 S Hanley Road, St Louis, MO 63144, USA). A constant dosing interval (tau) of 48 h was assumed for the calculation of steady-state AUC_0–48 h_ using the linear up/log down trapezoidal method. To normalize AUCs obtained with different silymarin doses, AUCs were divided by the milligram dose administered (AUC ng·h/mL·mg). All pharmacokinetic parameters are reported as medians and their first and third quartiles.

The metabolic ratio, MR_0–48 h_, was calculated as the AUC_0–48 h_ for total flavonolignan glucuronide and sulfate conjugates divided by the AUC_0–48 h_ of the parent flavonolignan. The metabolic ratio, MR_0-Tmax-P_, was calculated as the AUC_0-Tmax-P_ for total flavonolignan glucuronide and sulfate conjugates divided by the AUC_0-Tmax-P_ of the parent flavonolignan, where the interval, 0-Tmax-P, was defined as time 0 to the time in hours to reach the C_max_ for the parent flavonolignan.

### 3.9. Statistical Analysis

The association of *UGT1A1* polymorphism with the pharmacokinetics of SA and SB was examined from the comparison of the following measures of parent flavonolignan exposure according to UGT1A1*28 genotype: (1) the AUC_0–48 h_; (2) the MR_0–48 h_; and (3) the MR_0-Tmax-P_. A one-way analysis of variance was conducted using Kruskal-Wallis test to assess difference in parent flavonolignan exposures between *UGT1A1* genotypes, *p* < 0.05 significant (SAS JMP 6.0.0; SAS Institute, Inc., Cary, NC, USA). Student *t*-test were used for other statistical comparison analysis, *p* < 0.05.

## 4. Discussion

Silymarin is a milk thistle extract commonly used and studied for the treatment of chronic liver diseases both as in single agent and in combination with other natural products. However, conflicting results from numerous clinical studies raise questions regarding silymarin’s efficacy even though in vitro experiments suggest it has significant antioxidant, anti-inflammatory, and antifibrotic effects. The influence of genetic polymorphism on the metabolism and transport of xenobiotics contributes to large inter-individual differences in drug exposures and clinical outcomes. Silymarin undergoes extensive metabolism to glucuronides following oral dosing and UGT1A isoforms (i.e., UGT1A1, 1A3, 1A8 and 1A10) have been demonstrated to play a major role in the glucuronidation of silymarin flavonolignans in vitro [17]. Since large inter-individual differences in the glucuronidation of silymarin may confound the assessment of silymarin’s efficacy in humans, we evaluated the relationship between *UGT1A1* polymorphism and the metabolism and pharmacokinetics of silymarin in patients with liver disease.

The frequency of the homozygous UGT1A1 *28/*28, heterozygous UGT1A1 *1/*28, and wild-type UGT1A1 *1/*1 genotypes were similar to those previously reported [31]. Our in vitro enzyme kinetic studies with UGT1A1 indicate a high intrinsic clearance for SB, and since approximately 71% of SB conjugates found in plasma are glucuronides [30], we expected the concentrations of SB glucuronides to be lower in patients carrying the UGT1A1*28 allele. However, no significant differences were observed in the AUC_0–48 h_ for either SA or SB among *UGT1A1* genotypes. Similarly, no significant differences in MR_0–48 h_ and MR_0-Tmax-P_, metabolic ratios that account for differences in the extent of conjugation and enterohepatic cycling, respectively, were observed among the three genotypes. Thus, the UGT1A1*28 polymorphism does not appear to effect silymarin exposures in patients with chronic HCV infection.

In this study, we observed lower metabolic ratios in patients with NAFLD compared to those with HCV infection which raises the possibility that the influence of liver disease may mask the influence of UGT1A1*28 polymorphism on the pharmacokinetics of silymarin. The factors contributing to differences in flavonolignan exposures between HCV and NAFLD patients are unclear. However, reduced hepatic UGT mRNA in inflamed tissue [32] and altered hepatic expression of some drug transporters, such as down-regulated MRP2 and up-regulated MDR1, MRP1, and MRP3 in livers of patients with HCV infection may be associated with differences in the pharmacokinetics of silymarin between patients with NAFLD and HCV chronic infection. Elevated serum bilirubin has been associated with the UGT1A1*28 allele. However, no relationship was found between total bilirubin levels and UGT1A1*28 polymorphism in our study. Many factors influence serum bilirubin levels such as smoking status, fasting status, diseases, especially diseases of the liver, which may have obscured the effects of UGT1A1*28 polymorphism. UGT1A1 has overlapping substrate specificity for bilirubin and silymarin flavonolignans, and to some degree, bilirubin and flavonolignan conjugates are both transported across the canalicular membrane into bile by the apical conjugate export pump, MRP2. However, we did not observe an association between flavonolignan metabolic ratios and bilirubin in patients within the same *UGT1A1* genotype group which suggests the influence of UGT1A1*28 polymorphism on silymarin metabolism in HCV-infected or NAFLD patients may be masked by a greater effect of disease state.

To further examine the effects of UGT1A1*28 polymorphism on the metabolism of silymarin, we first identified the glucuronide profile for each of the six silymarin flavonolignans following the incubation of each flavonolignan with recombinant UGT1A1. Only a few studies have been carried out on the metabolism of silymarin because of the multiple phenolic and hydroxyl sites for conjugation. Two main silybin A and silybin B glucuronides (7- and 20-β-d-glucuronides) have been reported [33]. In our in vitro studies with UGT1A1, the 7-β-d-glucuronide was the major metabolite for both silybin A and silybin B which is similar to the findings of Kren et al. [34] who suggested greater glucuronidation at the C-7 position may be due to hydrogen-bonding with an adjacent C_19_ methoxy group. In contrast, we observed the 20-β-d-glucuronide was the major silybin B conjugate in human plasma which suggests differences in the stereoselective hepatic uptake or efflux of silybin B glucuronides into bile may be responsible for relatively higher proportion of the silybin B 20-β-d-glucuronide in plasma.

With the exception of silybin A, glucuronide profiles were similar when silymarin flavonolignans were incubated with either human liver microsomes or with recombinant UGT1A1 indicating UGT1A1 is a major UGT isozyme in the glucuronidation of silymarin flavonolignans. Since the C-20 silybin A glucuronide was only formed during incubations with HLMs but not with UGT1A1, we conclude UGT1A1 is not a major contributor to the glucuronidation of silybin A at the C-20 position. Five distinct glucuronide peaks were identified in the pooled plasma from subjects with different UGTIA1*28 genotypes using HLM generated glucuronide profiles for each silymarin flavonolignan as reference samples. UGT1A1*28 polymorphism had no effect on the profile of flavonolignan glucuronides detected in plasma. Since other UGT isozymes (i.e., UGT1A6, 1A7, 1A9, 2B7 and 2B15) have been shown to participate in the glucuronidation of silymarin [17], compensatory up-regulation of other UGT isozymes in patients with reduced UGT1A1 may mask an effect of the UGT1A1*28 polymorphism on silymarin exposure. For example, Wang et al. observed similar or higher glucuronidation of flavonoids in UGT1A-deficient Gunn rats compared with Wister rats due to the up-regulation of UGT2Bs [35].

In our study, sulfate conjugates represented approximately 17% of the total flavonolignan conjugates found in the plasma from patients with HCV infection or NAFLD which is similar to what has been previously reported in healthy volunteers. Data obtained in Gunn rats suggest an increase in the sulfation of silymarin flavonolignans, especially silybin A and silybin B, in the absence of UGT1A isoforms, and these sulfate conjugates appear to be more readily excreted into the bile [18]. Therefore, we evaluated whether the presence of UGT1A1*28 allele was associated with an increase in the sulfation of silymarin flavonolignans which might mask effects on endpoints that are based on total conjugates such as in our calculation of metabolic ratios. Interestingly, a slight decrease in the proportion of flavonolignan glucuronide conjugates and an increase in the proportion of sulfate conjugates was observed in pooled plasma from subjects with a UGT1A1*28/*28 genotype compared to subjects carrying wild type alleles. More favorable efflux of flavonolignan sulfates into bile compared to flavonolignan glucuronides could account for just the slight increase in flavonolignan sulfates detected in the plasma of subjects enrolled in this study.

## 5. Conclusions

In summary, our results indicate the UGT1A1*28 allele has no significant effect on the pharmacokinetics of silybin A and silybin B in patients with NAFLD or chronic HCV infection. Increased sulfate conjugation may partly compensate for reduced glucuronidation so that total flavonolignan metabolism and plasma exposures of parent flavonolignans are unchanged. Therefore, we conclude it is unlikely the presence of the UGT1A1*28 allele may have confounded the detection of silymarin’s efficacy in previous well-designed randomized controlled trials such as the SyNCH phase IIb study [14]. Other factors that may be responsible for the large inter-subject variability in the pharmacokinetics of silybin A and silybin B which may be obscuring the ability to detect the beneficial effects of silymarin in patients with liver disease include: variability in the dissolution and absorption of silymarin in the gut as has been demonstrated in animals and humans; variability in the extent of enterohepatic recycling of flavonolignans; and the effects of liver disease on mechanisms that govern the disposition of silymarin in man, which were briefly discussed above [15,36,37].

## Figures and Tables

**Table 1:** UDP-Glucuronosyltransferase 1A1 Michaelis–Menten kinetic parameters for flavonolignans by substrate depletion method.

Flavonolignan	*K_m_* (μM)	*V_max_* (pmol/mg/min)	*Cl_int_* (μL/mg/min)
Silybin A	23.7 ± 17.7	2610 ± 1500	110.0
Silybin B	8.7 ± 0.8	5920 ± 4550	691.0
Silydianin	9.0 ± 0.8	12,740 ± 3710	1411.0

Expressed recombinant human UGT1A1 was pretreated with alamethicin (50 μg/mg protein) and incubated with varying concentrations of SA or SB or silydianin to determine the enzyme kinetic parameters as described in Materials and Methods. The data were fitted to Michaelis-Menten kinetics to obtain apparent *K_m_* values. Data are expressed as mean ± S.D (*n* = 3).

**Table 2:** Demographic characteristic of subjects in the three study groups based on genotype of UGT1A1.

Demographics and Laboratory Values	UGT1A1 Genotype		
	*1/*1	*1/*28	*28/*28
Number of subjects	10	14	5
Male:Female	3:7	8:6	4:1
HCV:NAFLD	6:4	10:4	3:2
White:Black:Hispanic	9:1:0	13:0:1	1:4:0
Age (year) ^a^	51.4 (27.8, 60.6)	50.5 (43.0, 57.2)	48.7 (43.1, 55.9)
BMI (kg/m^2^) ^a^	28.9 (21.1, 42.4)	29.5 (21.5, 36.6)	34.8 (25.7, 39.2)
Total Bilirubin (mg/dL) ^a^	0.45 (0.1, 1.1)	0.65 (0.3, 0.8)	0.7 (0.3, 2.6)
Direct Bilirubin (mg/dL) ^a^	0.29 (0.1, 0.8)	0.26 (0.1, 0.6)	0.47 (0.3, 0.6)
ALT (U/L) ^a^	88.5 (48, 164)	94 (58, 322)	79 (52, 158)
Platelet Count (cells/mm^3^) ^a^	228.5 (139, 339)	194.5 (162, 319)	254 (108, 327)

^a^ Data presented as median (minimum, maximum) values.

**Table 3:** Silybin A and Silybin B dose-normalized exposures (AUC_(0–48 h)_, ng·h/mL⋅mg) in patients with different *UGT1A1* genotypes.

Flavonolignan	UGT1A1*1/*1 (*n* = 10)	UGT1A1*1/*28 (*n* = 14)	UGT1A1*28/*28 (*n* = 5)	*p*-Value
	Median (25th, 75th)	Median (25th, 75th)	Median (25th, 75th)	*1/*1 vs. *1/*28 vs. *28/*28
Silybin A	0.17 (0.13, 0.2)	0.19 (0.15, 0.23)	0.12 (0.1, 0.23)	0.78
Silybin B	0.02 (0.02, 0.03)	0.02 (0.01, 0.03)	0.02 (0.02, 0.04)	0.76

## Abbreviations

SC, silychristin; SD, silydianin; SA, silybin A; SB, silybin B; ISA, isosilybin A; ISB, isosilybin B; HCV, hepatitis C virus; WT, wild-type phenotype, UGT1A1-competent; CAMs, complementary and alternate medicines; LC-ESI-MS, liquid chromatography-electrospray ionization-mass spectrometry; AUC_0→48h_, area under the plasma concentration-time curve from time 0 to 48 h; AUC_0→tmax_, area under the plasma concentration-time curve from time 0 to time of maximum concentration.
